# Assessing the Evidence for Differential Health Effects of Wildfire Smoke Across Fires and Populations: A Critical Review of Recent Studies

**DOI:** 10.1007/s40572-026-00548-4

**Published:** 2026-06-30

**Authors:** Colleen E. Reid, Sheryl Magzamen

**Affiliations:** 1 Geography Department and Institute of Behavioral Science, University of Colorado Boulder, Boulder, CO 80309 USA; 2 Department of Environmental and Radiological Health Sciences, Colorado State University, Fort Collins, CO 80523 USA

**Keywords:** Wildfires, PM_2.5_, Wildfire smoke, Public health

## Abstract

**Purpose of review:**

To evaluate the recent literature on differential exposure to and health risks from wildfire smoke across subpopulations, whether wildfire-derived PM_2.5_ affects health differently from non-wildfire-derived PM_2.5_, and how wildfire smoke composition affects health.

**Recent Findings:**

We found inconsistent evidence of differential exposure to and health risks from wildfire PM_2.5_ by population subgroups. This could be due to variation in wildfire PM_2.5_ infiltration into buildings and ability to take individual protective actions, both of which have been noted to be related to socio-economic status in the recent scientific literature. Respiratory health endpoints have been the most consistent and commonly evaluated health outcome in studies of wildfire smoke; additional research is needed to resolve conflicting findings for non-respiratory health outcomes (e.g., cardiovascular disease). Although some recent studies have documented larger health risks from wildfire-derived as compared to non-wildfire-derived PM_2.5_, we document how further research could evaluate whether these findings are confounded by type of fuel burned, due to methodological concerns, or are true. We also conclude that more research is necessary to elucidate potential differences in health risks of constituents of wildfire smoke other than PM_2.5_ or from burning of different fuels.

**Summary:**

Wildfire smoke is projected to continue to increase. We encourage future research to move away from further documentation of respiratory health impacts of wildfire smoke, which has been very well established, into studies of other health endpoints that have been less well studied to date, more exploration into health effects from wildfire smoke constituents other than PM2.5 and from different types of fires (i.e., wildland urban interface (WUI) fires versus wildland fires), and additional exploration of remaining uncertainties with a goal of further supporting public health protection from wildfire smoke.

## Introduction

**Fig. 1:**
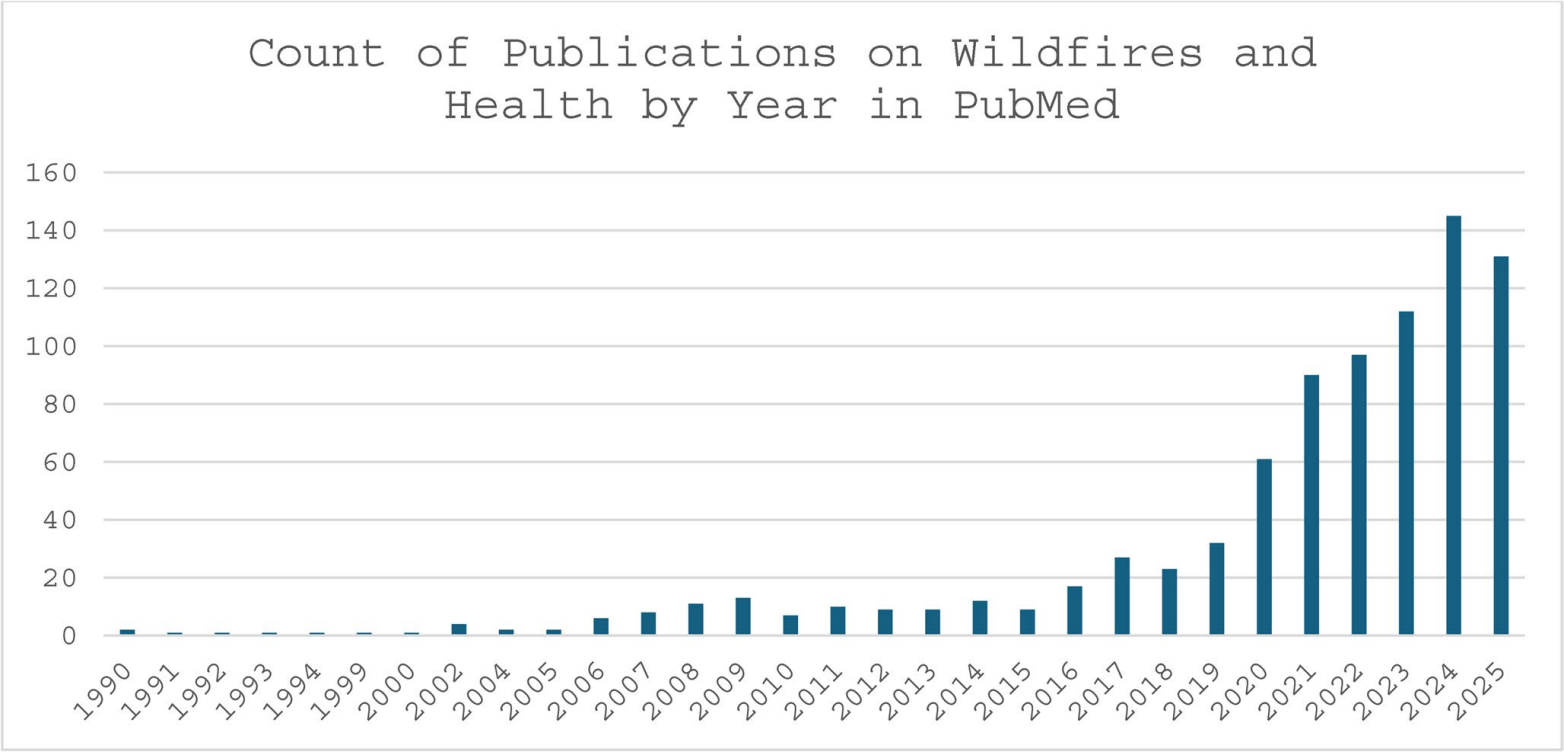
Count of articles indexed in PubMed as of October 15, 2025 by year that have in their title or abstract the word “smoke” and the word “health” and one of the following: wildfire, wildland fire, forest fire, bushfire, grassland fire, peat fire, boreal fire, prescribed fire, WUI fire, agricultural burn, or landscape fire

There is growing interest in understanding the health effects of wildfire smoke exposure globally, and specifically within the U.S. Over the previous five decades, the Western U.S. has experienced increases in wildfire occurrence, total area burned, length of wildfire season, and fire size [1–5]. The smoke resulting from these fires, a complex mixture of gases and airborne particles, has reversed decades of decline in anthropogenic fine particulate matter concentrations (PM_2.5_; particles with an aerodynamic diameter less than or equal to 2.5 μm) with the most prominent increases occurring in regions of the western US [4, 6, 7]. As wildfire smoke can travel far distances, smoke exposure is not only a health concern for people who live directly downwind of the fire but also for those who live thousands of kilometers from the fire source [8] and across national borders [9]. The overall increase in wildfire smoke events has likely contributed to the exponential increase in scholarly investigation over the last decade into the health effects of wildfire smoke (Fig. 1).

Epidemiologic studies have consistently demonstrated health effects in response to short-term (i.e., over one or a few days) wildfire smoke exposure on numerous respiratory-related effects, specifically exacerbations of asthma and chronic obstructive pulmonary disease (COPD) resulting in emergency department visits and hospital admissions [10–13]. More recently, however, studies have provided initial evidence of relationships between short-term wildfire smoke exposure and a range of other health effects such as impacts on brain function [14], pre-term birth [15], atopic dermatitis [16], blood pressure [17], cardiac arrest mortality [18], exacerbation of kidney disease [9, 19], and emergency department visits for mental health conditions [20, 21].

While epidemiologic studies have primarily focused on short-term smoke exposures, wildfires are no longer rare, singular events, but occur in many locations multiple times during a year or every year. As a result, some recent studies have explored health implications of longer-duration smoke exposures occurring multiple times within and across years. These studies have provided initial evidence that longer-duration smoke exposures are associated with increased risk of influenza [22], incident dementia [23], heart failure [24], cancer mortality [25], and mortality [26]. Most of these studies, however, have used exposure metrics that represent long-term average exposure, which ignores the highly dynamic nature of smoke exposures. Recent commentaries have therefore highlighted the need to account for the dynamism of smoke exposures by investigating exposure metrics that evaluate the frequencies, intensities, and durations of exposures [27, 28].

Wildfire smoke, as a source of air pollution, cannot be controlled through traditional technologies implemented through regulatory actions, such as have been implemented for point sources (e.g., industrial facilities) and mobile sources (e.g., internal combustion engines) of PM_2.5_. In contrast, the protection of public health revolves around avoidance and reduction of wildfire smoke exposures through individual- and community-level action. Considering the proliferation of publications on wildfire smoke and health, now is an opportune time to assess the current literature, focusing on recent novel developments in wildfire smoke epidemiology and exposure assessment. This review focuses on studies published between 2020 and 2025 that provide insights into the following topics: (1) differential exposure to and health risks of wildfire smoke on population subgroups, (2) whether there is evidence of differential health risks between PM_2.5_ derived from wildfire smoke or from other sources, and (3) the health effects associated with specific components of wildfire smoke. Additionally, we identify gaps in published literature with respect to these topics that, if explored, could further support the protection of public health from this growing source of air pollution.

## Examining Differential Exposure and Health Risks of Wildfire Smoke

### Is There Differential Exposure to Wildfire Smoke By Demographic Characteristics?

Understanding patterns of wildfire smoke exposure is important in the context of ensuring that public health actions (e.g., location of clean air centers, air cleaner distribution programs, N95 mask distribution) during smoke events focus on those most affected. Recently, some studies have examined whether specific populations are disproportionately exposed to wildfire smoke. In a national analysis examining patterns of wildfire smoke exposure from 2006 to 2020, Childs et al. investigated differential exposure to daily wildfire PM_2.5_ and found the highest exposures among Hispanic and non-Hispanic White populations [29]. Another national analysis by Rice et al., encompassing the years 2007–2018, found that Black Americans and Native Americans and American Indians had the highest overall exposure to wildfire smoke [7]. Both Rice et al. [7] and Childs et al. [29] examined whether there were differences in smoke exposure by income with Rice et al. concluding no observed differences in exposure and Childs et al. finding increasing exposures for all income categories over time with some evidence of the highest exposure for those in the highest income category. While the previous two studies examined differential exposure to *daily* wildfire smoke, another study conducted in California during the years 2006–2022 by Casey et al. focused on differential exposure to longer duration smoke exposures finding higher smoke exposures in the Native American and Alaska Native and non-Hispanic White populations compared to other groups [28]. While there is some concordance in these findings, they are not completely consistent across studies. Differences in the geographic scope, years covered in the analysis, which population groups were analyzed, and the exposure models used (i.e., relying on wildfire-specific or total PM_2.5_) could be reasons for the differences in findings across studies.

### Are There Differential Health Risks of Wildfire Smoke By Demographic Characteristics?

Despite studies reporting higher exposures to wildfire smoke for specific populations by race/ethnicity or income, observed differences in exposure are not consistently in alignment with differential health risks to wildfire smoke by these demographic groups in epidemiologic studies. In a study focusing on the 2008 northern California wildfires, Reid et al. (2023) found differential associations between PM_2.5_ and COPD exacerbations with the largest associations for ZIP codes with lower socioeconomic status (SES) despite not finding evidence of consistent differential exposure to wildfire smoke PM_2.5_ by those same measures of SES by ZIP code [30]. Letellier et al. (2025) also focused on a single wildfire season in California (i.e., November 2018) but reported no evidence of differences in the association between wildfire PM_2.5_ and respiratory hospitalizations by county-level measures of race, despite finding higher exposures to wildfire smoke in counties with more White residents [31]. While Reid et al. and Letellier et al. focused on specific years of fire activity, Stowell et al. (2025) examined whether there was evidence of differential associations between wildfire PM_2.5_ exposure and ED visits in California across multiple wildfire seasons (2012–2019). The authors reported generally consistent risk estimates for wildfire smoke on respiratory ED visits across levels of the CDC’s Social Vulnerability Index, indicating no differential impacts by this measure. When examining associations by race/ethnicity, however, Stowell et al. reported the largest association between wildfire PM_2.5_ exposure and respiratory ED visits for Black individuals, with no evidence of a difference in associations between White, Hispanic, or Asian/Pacific Islanders [32]. Do et al. (2024) also examined multiple wildfire seasons in California, but for a longer time period (2006–2019) and with a focus on respiratory acute care visits. Across this time period, the authors reported larger risk estimates in communities with the highest percent Black or Pacific Islanders, and lower risk estimates in communities with higher automobile ownership, a proxy for SES [33]. Further, in a study examining cardiorespiratory hospitalization risk associated with medium-term (3-month average, not daily) wildfire smoke PM_2.5_ across 15 states, associations between asthma and pneumonia hospitalizations, separately, with wildfire PM_2.5_ were found to be higher in areas with higher area deprivation indices [34]. These last three studies did not report if they analyzed for differential exposure. We know of no studies that have examined differential health risks of wildfire smoke exposure by demographic groups for health endpoints that are not respiratory in nature, thus more research is needed to fully understand differential health risks of wildfire smoke exposure by race/ethnicity and SES.

### What May Explain the Differential Health Risks of Wildfire Smoke?

Notably, these differences in health risks from short- and medium-term wildfire smoke exposure by racial categorization or measures of SES are not consistent across studies, which could be due to multiple factors including which measures of SES or categorizations of race/ethnicity were used, the exposure model used, the underlying susceptibility of the population, whether the study used individual-level or area-level data (and at what spatial aggregation), or other modeling choices. If there truly are differential health risks of wildfire smoke by SES or race/ethnicity, these could be due to differential susceptibility to wildfire smoke which could be due to higher levels of pre-existing conditions in these subpopulations, or due to misclassification of exposure.

Misclassification of exposure by SES or race/ethnicity could be due to numerous pathways. Exposure to wildfire smoke relies on models that use in situ monitors to ‘ground truth’ data. These exposure models thus perform best in areas with densely placed monitors, which are often urban areas [35]. Although the use of low-cost air quality sensors like Purple Air monitors [36] continues to grow and has been instrumental in filling in the spatial gaps in the regulatory monitoring network, particularly in rural areas in the United States [37] and to estimate PM_2.5_ during wildfire events [38, 39], these sensors tend to be found in densely populated areas with relatively high socioeconomic status and less racial and ethnic diversity [40, 41]. Thus, estimation of outdoor PM_2.5_ concentrations is not uniformly accurate across space, likely better representing exposure in areas with more monitors which could be higher SES areas. Comparisons of wildfire PM_2.5_ exposure models show inconsistencies in when and where different wildfire smoke exposure models perform best [42–44]. As a result, it is not clear which exposure models are optimal to employ in epidemiologic studies despite evidence that associations between wildfire smoke exposure and health endpoints differ when using different exposure models [45, 46].

Perhaps more importantly, epidemiologic studies to date likely misclassify exposures by relying solely on outdoor wildfire PM_2.5_ concentrations when we know that individuals, particularly in the US, spend most of their time indoors [47]. There is substantial evidence of differential levels of infiltration of PM_2.5_ into homes and commercial facilities that is due to building-level characteristics [48, 49] such as building age and the presence of an HVAC system. Epidemiologic studies have also demonstrated that air conditioning can modify the effects of outdoor concentrations of wildfire smoke on health risks [32, 33, 50]. It has been shown that AC prevalence is correlated with area-level measures of percent Black population [51, 52], percent Hispanic population, lower income, and lower educational attainment [52]. As a result, there could be differential indoor exposures during wildfire events by race/ethnicity or SES that are not captured by relying on only outdoor PM_2.5_ exposure assessment [53]. A recent publication estimates that this inaccuracy of exposure assessment, by relying solely on outdoor wildfire smoke exposure, may bias associations between wildfire PM_2.5_ and health outcomes towards the null [39].

Another aspect of exposure assessment that most epidemiologic studies do not account for are behaviors taken to protect oneself from wildfire smoke that could be differential by population subgroups. Magzamen et al. (2021) found wildfire smoke from *local* fires was associated with reduced odds of respiratory hospitalizations, but smoke from *distant* fires was associated with increased odds of respiratory hospitalizations [18]. The authors hypothesized that during local smoke events, people were more likely to shelter in place, or, in extreme cases, evacuate than they would for long-range transported smoke events. Both of these actions would decrease exposure to smoke and thus mitigate smoke-related health effects. A study that used multiple sources of information to understand population-level behaviors during wildfire smoke events found that online searches related to actions to protect health during wildfire smoke events were higher in wealthier areas, and, based on GPS data, that people in wealthier areas were more likely to stay completely at home during high smoke days as compared to people living in lower income areas [54]. As demonstrated during the COVID-19 pandemic, individuals of lower SES may be less likely to shelter in place due to job demands [55]. Thus, exposure misclassification could also occur due to differential ability to take protective measures, which could lead to differential health risks of wildfire smoke exposure.

## Comparison of Health Effects From Wildfire-derived PM_2.5_ Versus PM_2.5_ Emitted From Other Sources

Fine particulate matter (PM_2.5_) has been the primary exposure indicator used in wildfire smoke epidemiologic studies to date. This focus can be attributed to PM_2.5_ being a main component of wildfire smoke along with the well-documented evidence of health effects in response to exposure to PM_2.5_ from anthropogenic sources (e.g., industrial operations, vehicles) [56]. Building off extensive efforts to examine potential differences in the health risks posed by total PM_2.5_ emitted from different sources [57, 58], many recent epidemiologic studies have attempted to answer the question of whether PM_2.5_ from wildfire smoke affects health more or less than PM_2.5_ from other sources.

Up until 2021, most studies focused on documenting health effects associated with PM_2.5_ exposure during wildfires rather than trying to discern if wildfire-derived PM_2.5_ affected health to a greater or lesser degree than other sources of PM_2.5_. An epidemiological study published that year, however, suggested that differences in risk estimates for respiratory-related hospital admissions for wildfire-derived PM_2.5_ and PM_2.5_ from other sources indicated a difference in toxicity of PM_2.5_ in wildfire smoke [59]. In stating a “difference in toxicity,” those authors were referring to a difference in effect estimates for respiratory hospitalizations for wildfire-derived PM_2.5_ as compared to non-wildfire PM_2.5_ based on the results from models that demonstrated the most difference in effect estimates across many models reported in their study. Since that manuscript was published, however, many studies have aimed to conduct similar comparisons of associations between wildfire-derived and non-wildfire PM_2.5_ for a variety of health effects and outcomes. In the context of epidemiologic studies, asking whether PM_2.5_ from different sources differentially affects any health outcome is challenging because other factors could be contributing to the differences observed, such as demographic factors and overall differences in the concentration of the exposure, given that most wildfire smoke epidemiologic studies analyze data not just across time but also space. An additional complexity around such a comparison of wildfire-derived and non-wildfire PM_2.5_ is the fact that people are not exposed to one type of PM_2.5_ or another, but PM_2.5_ from all types of sources at once.

A main challenge in comparing health risk estimates between the different types of PM_2.5_ is that wildfire-derived PM_2.5_ exposure concentrations have a very different distribution than non-wildfire-derived PM_2.5_, with predominantly zero values on all days not affected by wildfire smoke and very high values on days with wildfire smoke, creating a zero-inflated right skewed distribution. As a result, the traditional approach of calculating risk estimates using a uniform exposure increment (i.e., interquartile range [IQR] or standardized value [such as 10 µg/m^3^]) becomes more challenging for a wildfire-derived PM_2.5_ exposure because the standardized values may not necessarily represent realistic increases in concentrations people actually experience during the smoke event.

One additional challenge of the comparison of health effects of wildfire-derived PM_2.5_ as compared to non-wildfire derived PM_2.5_ is that inhalable PM_2.5_ is emitted from multiple sources. Thus, it is important that studies adjust for these other sources of PM_2.5_ in their epidemiologic models because those other sources could be impacting the same health outcomes being analyzed. Additionally, people living in areas with higher background concentrations of PM_2.5_ from a variety of sources may be differentially affected by the addition of wildfire PM_2.5_ than people living in areas with lower background PM_2.5_ concentrations. Given that most epidemiologic studies of wildfire-derived PM_2.5_ compare exposures and health outcomes not just across time but also across space, adjustment for confounding by, or effect modification by, non-wildfire-derived PM_2.5_ is warranted.

To date, in the studies that compare health risks between wildfire-derived PM_2.5_ and non-smoke-derived PM_2.5_, most find larger risk estimates for wildfire-derived PM_2.5_, whether mutually-adjusted for non-wildfire-derived PM_2.5_ or not, particularly when using standardized increments (e.g., 1–10 µg/m^3^). This has been found for studies of short-term (daily to weekly) exposures on respiratory hospitalizations in California [59], acute respiratory infections in low and middle-income countries [60], diabetes hospitalizations across numerous world regions [61], and for longer-term exposures on incident dementia [23]. However, this is not always the case, particularly when using different standardized increments. One study that conducted sensitivity analyses where results were also presented using an IQR-based increment found similar associations between wildfire-derived and non-wildfire-derived PM_2.5_ and incident dementia [62]. Additionally, a study conducted in New York City during the 2023 Canadian fire smoke period reported differences in associations depending on the health outcome examined, with wildfire-derived PM_2.5_ being more strongly associated with increases in ED visits for asthma and COPD but non-wildfire PM_2.5_ more strongly associated with cardiovascular ED visits [63].

## Comparison of Health Effects From Other Components of Wildfire Smoke Beyond PM_2.5_

Wildfire smoke is not solely made up of PM_2.5_, and smoke from different types of fires (e.g., agricultural fires, prescribed fires, boreal fires, wildland-urban interface [WUI] fires) could have different chemistry and subsequently different effects on health. Some studies have shown that aged smoke is chemically different from “fresh” smoke [64, 65], with hazardous air pollutants like benzene, formaldehyde, and acetaldehyde in greater abundance near the site of the fire. Fires also have flaming and smoldering periods, which may lead to differential smoke toxicity [66]. Additionally, a study that investigated differential health risks of PM_2.5_ from fires that burned different fuels found the strongest association with asthma ED and urgent care visits from fires that burned predominantly forests whereas smoke from shrub/scrub fires had a stronger influence on dysrhythmias [67]. Additionally, wildfires that enter the WUI burn human-made materials, such as vehicles and structures, in addition to vegetation. Human-made materials when burnt emit different compounds that may be more toxic than purely biomass-based smoke [68, 69]. As there are more instances of wildfires entering the WUI recently [70], more work is needed to evaluate the health impacts of smoke exposure from WUI fires as compared to fires that burn predominantly vegetation.

Very few epidemiologic studies have investigated other components of wildfire smoke besides PM_2.5_. Studies show that wildfire smoke includes numerous VOCs [71] that have the potential to affect health. VOCs can also influence the formation of ground-level ozone [72] which is a respiratory irritant and has been associated with mortality and other health outcomes [73]. Very few studies have examined health risks due to ozone formed downwind of a fire. Chen et al. (2024) found short-term exposure to ozone due to wildfire smoke was associated with increases in all-cause and respiratory, but not cardiovascular, mortality in a global study [74], and Reid et al. (2019) found that ozone during a wildfire episode in California in 2008 was associated with respiratory ED visits but upon adjustment for PM_2.5_, associations with ozone were null [75]. While it would not be expected that ozone formed from precursors emitted during wildfires would affect health differently from ozone formed through emissions from other polluting sources, understanding the health risks of ozone from wildfires is an understudied area that could be further explored, particularly when thinking about the mixture of exposures in wildfire smoke. Although there is much interest in the potential differential health risks of wildfire-derived PM_2.5_ based on chemical composition, as of yet, studies have not examined the combined health effects of all individual pollutants within wildfire smoke.

## Discussion

The number of studies examining the health effects attributed to wildfire smoke has grown substantially over the past five years. These studies show a clear and consistent relationship between short-term wildfire smoke exposures and various health effects. This review of recent studies highlights some new and emerging themes within that work that warranted discussion and critical evaluation, particularly as they relate to novel health endpoints evaluated, whether there are differential health risks by SES or race/ethnicity, and whether other components of wildfire smoke besides PM_2.5_ affect health. During the past five years, there has also been a large exploration into differential health effects of wildfire-derived PM_2.5_ as compared to non-wildfire PM_2.5_ that we thought deserved exploration. From a perspective of informing health protective actions for wildfire smoke exposure in a changing climate, we think this line of inquiry may miss the mark.

Some new health endpoints have been evaluated for their relationship with wildfire smoke exposure over the past five years including impacts on brain function [14], pre-term birth [15], atopic dermatitis [16], blood pressure [17], cardiac arrest mortality [18], exacerbation of kidney disease [9, 19], and emergency department visits for mental health conditions [20, 21]. At this point in time, however, there has not been enough research into these new health impacts of wildfire smoke to assess whether the weight of the evidence indicates a consistent association. Additionally, more papers have recognized that wildfire smoke events are becoming more frequent and thus, there is a need to investigate health effects from repeated exposures to wildfire smoke as well as to explore impacts on health effects that have a longer latency than a few days to a few weeks. To date, however, not enough research has been done on repeated exposures to wildfire smoke and health impacts that occur with longer latency times .

Across the studies evaluated, findings of differential exposure to wildfire smoke by SES or race/ethnicity are inconsistent and any observed differential health risks of wildfire smoke by SES or race/ethnicity do not always align with differential exposure in these studies. Inconsistency in findings between studies and differential health risks without differential exposures by the same population groups within a study could be due to numerous factors. In this review, we highlight the potential role of exposure misclassification. We believe exposure misclassification could be a significant factor due to the large number of studies that have documented differential performance of exposure models in areas with and without monitoring data, reliance on estimates of only outdoor smoke exposure when there is evidence of differential infiltration of wildfire smoke into buildings, and differential abilities of individuals to take protective behavioral measures to reduce smoke exposure during wildfire smoke events.

Within this review, we document the growing number of epidemiologic studies that posit that the magnitude of health effects associations with wildfire-derived PM_2.5_ are larger than those with non-wildfire PM_2.5_. While many of the more recent studies making this comparison do mutually adjust for non-wildfire PM_2.5_, which we think is important given that people inhale PM_2.5_ from multiple sources, we posit that there are other methodological challenges in assessing the health risks of these two sources of PM_2.5_; this is a critical area for evaluation in future work, particularly the non-normal distribution of wildfire-derived PM_2.5_ exposures. Additionally, a larger fundamental question exists as to whether this research question is necessary. Ambient PM_2.5_ is a health hazard regardless of source. Wildfire smoke represents a source of air pollution that cannot be regulated like point and mobile sources of PM_2.5_ for which technological solutions exist. While changes to forest management such as increased forest thinning or prescribed fire attempt to decrease the risk of wildfires, to date we know of no evidence as to the degree of thinning and prescribed fire that would be needed to sufficiently decrease the risk of wildfires especially under current and future levels of climate change. Prescribed fires also emit PM_2.5_, which can also affect health. Thus, public health interventions such as promoting behavior changes at the individual level by staying indoors, using air cleaners, and wearing masks [76] are currently the best approach to protect the public’s health during wildfire smoke events. Given the fact that getting individuals to change their behavior can be difficult, another way to protect people’s health from wildfire smoke would be to reduce the sources of non-wildfire PM_2.5_ which has proven to be effective through actions taken under the Clean Air Act [77].

Wildfire smoke is not homogeneous and what burns during a wildfire can affect the chemistry of the PM_2.5_ emitted as well as the gases in the smoke plume. To date there has been little research into the health effects of other air pollutants found within wildfire smoke (e.g., VOCs) or formed by precursors within smoke (e.g., ozone). Additionally, it remains unclear how wildfires that burn different fuels, such as forests, grasslands, peat, or structures, could influence health risks. Information obtained from future research into these topics could aid in elucidating whether more refined health messaging or interventions to reduce smoke exposures are needed for wildfires that burn different fuels or for long-range transported smoke.

## Conclusion

Given that the best efforts to decrease health risks from wildfire smoke known to date include individual-level behavior change, we propose that future studies focus on which populations are differentially harmed by wildfire smoke and interrogations into the reasons for those differential health risks. While there is preliminary evidence that this could be due to exposure misclassification by relying on outdoor concentrations of wildfire smoke, more work is needed to discern to what extent this is the case and what role differential susceptibility may also play. Findings on this topic can influence whether we focus on interventions to improve indoor air in the leakiest buildings or for the most susceptible groups. Due to methodological and practical concerns, the current focus on distinguishing between health risks of wildfire-derived versus non-wildfire-derived PM_2.5_ should be a lower priority research area, given that people are exposed to all PM_2.5_ not solely PM_2.5_ from wildfire smoke and due to the challenges in preventing wildfires especially as our climate is changing. The past five years of peer-reviewed scientific studies focused on the health impacts of wildfire smoke have provided significant insights. Further work into the unique health impacts of wildfire smoke, both in total, and from different fuels, could provide more evidence about where and when public health interventions could be most useful.

The authors report that they have no financial or non-financial competing interests with regards to this publication.

## Data Availability

No datasets were generated or analysed during the current study.

## References

[1] Cattau ME, Wessman C, Mahood A, Balch JK. Anthropogenic and lightning-started fires are becoming larger and more frequent over a longer season length in the U.S.A. Glob Ecol Biogeogr. 2020;29:668–81.

[2] Dennison PE, Brewer SC, Arnold JD, Moritz MA. Large wildfire trends in the western United States, 1984–2011. Geophys Res Lett. 2014;41:2928–33.

[3] Westerling AL. Increasing western US forest wildfire activity: sensitivity to changes in the timing of spring. Philos Trans R Soc Lond B Biol Sci. 2016. 10.1098/rstb.2015.0178. 27216510 10.1098/rstb.2015.0178 PMC4874415

[4] McClure CD, Jaffe DA. US particulate matter air quality improves except in wildfire-prone areas. Proc Natl Acad Sci U S A. 2018;115:7901–6. 30012611 10.1073/pnas.1804353115 PMC6077721

[5] O’Dell K, Ford B, Fischer EV, Pierce JR. Contribution of wildland-fire smoke to US PM2.5 and its influence on recent trends. Environ Sci Technol. 2019;53:1797–804. 30681842 10.1021/acs.est.8b05430

[6] Burke M, Driscoll A, Heft-Neal S, Xue J, Burney J, Wara M. The changing risk and burden of wildfire in the United States. Proc Natl Acad Sci U S A. 2021. 10.1073/pnas.2011048118. 33431571 10.1073/pnas.2011048118 PMC7812759

[7] Rice RB, Sacks JD, Baker KR, LeDuc SD, West JJ. Wildland fire smoke adds to disproportionate PM2.5 exposure in the United States. ACS EST Air. 2025;2:215–25. 40256491 10.1021/acsestair.4c00173 PMC12004501

[8] O’Dell K, Bilsback K, Ford B, Martenies SE, Magzamen S, Fischer EV, et al. Estimated mortality and morbidity attributable to smoke plumes in the United States: not just a Western US problem. Geohealth. 2021;5:e2021GH000457. 34504989 10.1029/2021GH000457 PMC8420710

[9] Song H, Liang M, Sieck NE, et al. The 2023 Canadian Wildfires and Risk of Hospitalization and Mortality Among Hemodialysis Patients in the United States. Kidney Int Rep. 2025;10:1750–60. 40630285 10.1016/j.ekir.2025.04.002 PMC12231018

[10] Reid CE, Brauer M, Johnston FH, Jerrett M, Balmes JR, Elliott CT. Critical review of health impacts of wildfire smoke exposure. Environ Health Perspect. 2016;124:1334–43. 27082891 10.1289/ehp.1409277 PMC5010409

[11] Cascio WE. Wildland fire smoke and human health. Sci Total Environ. 2017;624:586–95. 29272827 10.1016/j.scitotenv.2017.12.086 PMC6697173

[12] Gould CF, Heft-Neal S, Prunicki M, Aguilera J, Burke M, Nadeau K. Health effects of wildfire smoke exposure. Annu Rev Med. 2023. 10.1146/annurev-med-052422-020909. 37738508 10.1146/annurev-med-052422-020909 PMC12183787

[13] Black C, Tesfaigzi Y, Bassein JA, Miller LA. Wildfire smoke exposure and human health: significant gaps in research for a growing public health issue. Environ Toxicol Pharmacol. 2017;55:186–95. 28892756 10.1016/j.etap.2017.08.022 PMC5628149

[14] Cleland SE, Wyatt LH, Wei L, Paul N, Serre ML, West JJ, et al. Short-term exposure to wildfire smoke and PM2.5 and cognitive performance in a brain-training game: a longitudinal study of U.S. adults. Environ Health Perspect. 2022;130:67005. 35700064 10.1289/EHP10498 PMC9196888

[15] Picciotto S, Huang S, Lurmann F, et al. Pregnancy exposure to PM2.5 from wildland fire smoke and preterm birth in California. Environ Int. 2024;186:108583. 38521046 10.1016/j.envint.2024.108583 PMC11410054

[16] Fadadu RP, Grimes B, Jewell NP, Vargo J, Young AT, Abuabara K, et al. Association of wildfire air pollution and health care use for atopic dermatitis and itch. JAMA Dermatol. 2021. 10.1001/jamadermatol.2021.0179. 33881450 10.1001/jamadermatol.2021.0179 PMC8060890

[17] Walker ES, Stewart T, Vedanthan R, Spoon DB. Associations between fine particulate matter and in-home blood pressure during the 2022 wildfire season in Western Montana, USA. Environ Res Health. 2025;3:035002. 40416733 10.1088/2752-5309/add616 PMC12096407

[18] Magzamen S, Gan RW, Liu J, O’Dell K, Ford B, Berg K, et al. Differential cardiopulmonary health impacts of local and long-range transport of wildfire smoke. Geohealth. 2021;5:e2020GH000330. 35281479 10.1029/2020GH000330 PMC8900982

[19] Xi Y, Kshirsagar AV, Wade TJ, Richardson DB, Brookhart MA, Wyatt L, et al. Mortality in US hemodialysis patients following exposure to wildfire smoke. J Am Soc Nephrol. 2020. 10.1681/ASN.2019101066. 32675302 10.1681/ASN.2019101066 PMC7460895

[20] Jung YS, Johnson MM, Burke M, Heft-Neal S, Bondy ML, Chinthrajah RS, Cullen MR, Nelson L, Dresser C, Nadeau KC. Fine Particulate Matter From 2020 California Wildfires and Mental Health-Related Emergency Department Visits. JAMA Netw Open. 2025;8:e253326. 40184065 10.1001/jamanetworkopen.2025.3326 PMC11971671

[21] Zhu Q, Zhang D, Wang W, et al. Wildfires are associated with increased emergency department visits for anxiety disorders in the western United States. Nat Ment Health. 2024;2:379–87. 39568497 10.1038/s44220-024-00210-8 PMC11575985

[22] Orr A, Alden NB, Austin E, et al. Wildfire-season fine particulate matter exposure and associations with influenza and influenza-like-illness risk in the Western USA. Environ Health Perspect. 2025. 10.1289/EHP16607. 42564835 10.1021/EHP.6c00326 PMC13445268

[23] Zhang B, Weuve J, Langa KM, et al. Comparison of Particulate Air Pollution From Different Emission Sources and Incident Dementia in the US. JAMA Intern Med. 2023;183:1080–9. 37578757 10.1001/jamainternmed.2023.3300 PMC10425875

[24] Hao H, Xu K, Zhang D, Deng Y, Al-Kindi S, Pattisapu VK, et al. Long-term wildfire smoke exposure and increased risk of heart failure in older adults. J Am Coll Cardiol. 2025;85:2439–51. 40562508 10.1016/j.jacc.2025.04.058 PMC12367337

[25] Gao Y, Huang W, Xu Z, et al. Wildfire-related PM2.5 and cause-specific cancer mortality. Ecotoxicol Environ Saf. 2024;285:117023. 39278001 10.1016/j.ecoenv.2024.117023

[26] Gao Y, Huang W, Xu R, et al. Association between long-term exposure to wildfire-related PM2.5 and mortality: A longitudinal analysis of the UK Biobank. J Hazard Mater. 2023;457:131779. 37307727 10.1016/j.jhazmat.2023.131779

[27] Sacks JD, Migliaccio CT, Reid CE, Montrose L. Shifting the conversation on wildland fire smoke exposures: more smoke within and across years requires a new approach to inform public health action. ACS EST Air. 2025;2:122–9. 40182508 10.1021/acsestair.4c00236 PMC11964113

[28] Casey JA, Kioumourtzoglou M-A, Padula A, et al. Measuring long-term exposure to wildfire PM2.5 in California: time-varying inequities in environmental burden. Proc Natl Acad Sci U S A. 2024;121:e2306729121. 38349877 10.1073/pnas.2306729121 PMC10895344

[29] Childs ML, Li J, Wen J, Heft-Neal S, Driscoll A, Wang S, et al. Daily local-level estimates of ambient wildfire smoke PM2.5 for the contiguous US. Environ Sci Technol. 2022. 10.1021/acs.est.2c02934. 36134580 10.1021/acs.est.2c02934

[30] Reid CE, Considine EM, Watson GL, Telesca D, Pfister GG, Jerrett M. Effect modification of the association between fine particulate air pollution during a wildfire event and respiratory health by area-level measures of socio-economic status, race/ethnicity, and smoking prevalence. Environ Res Health. 2023;1:025005. 38332844 10.1088/2752-5309/acc4e1 PMC10852067

[31] Letellier N, Hale M, Salim KU, Ma Y, Rerolle F, Schwarz L, et al. Applying a two-stage generalized synthetic control approach to quantify the heterogeneous health effects of extreme weather events: a 2018 large wildfire in California event as a case study. Environ Epidemiol. 2025;9:e362. 39744585 10.1097/EE9.0000000000000362 PMC11692959

[32] Stowell JD, Sue Wing I, Romitti Y, Kinney PL, Wellenius GA. Emergency department visits in California associated with wildfire PM2.5: differing risk across individuals and communities. Environ Res Health. 2025;3:015002. 39670153 10.1088/2752-5309/ad976d PMC11632356

[33] Do V, Chen C, Benmarhnia T, Casey JA. Spatial heterogeneity of the respiratory health impacts of wildfire smoke PM2.5 in California. Geohealth. 2024;8:e2023GH000997. 38560560 10.1029/2023GH000997 PMC10978801

[34] Wei Y, Castro E, Yin K, Shtein A, Vu BN, Danesh Yazdi M, Li L, Liu Y, Peralta AA, Schwartz JD. Medium-term exposure to wildfire smoke PM2.5 and cardiorespiratory hospitalization risks. Epidemiology. 2025. 10.1097/ede.0000000000001881. 40433992 10.1097/EDE.0000000000001881 PMC12234148

[35] Miranda ML, Edwards SE, Keating MH, Paul CJ. Making the environmental justice grade: the relative burden of air pollution exposure in the United States. Int J Environ Res Public Health. 2011;8:1755–71. 21776200 10.3390/ijerph8061755 PMC3137995

[36] Barkjohn K, Gantt B, VonWald I, Clements A. (2019) PurpleAir PM2.5 U.S. Correction and Performance During Smoke Events 4/2020. https://cfpub.epa.gov/si/si_public_record_report.cfm?dirEntryId=349513&Lab=CEMM&simplesearch=0&showcriteria=2&sortby=pubDate&timstype=&datebeginpublishedpresented=08/25/2018. Accessed 24 Nov 2020.

[37] Sablan O, Ford B, Gargulinski E et al. Quantifying prescribed-fire smoke exposure using low-cost sensors and satellites: Springtime Burning in Eastern Kansas. GeoHealth. 2024; 8:e2023GH000982. 10.1029/2023GH000982 PMC10975953 38560558

[38] Raffuse S, O’Neill S, Schmidt R. A model for rapid wildfire smoke exposure estimates using routinely-available data - rapidfire v0.1.3. EGUsphere. 2023; 1–26. 10.5194/gmd-17-381-2024 PMC11469206 39398326

[39] Coker ES, Ho W, Paul N, Lee MJ, Dickson JM, Greif O, et al. Enhancing wildfire smoke exposure assessment: a machine learning approach to predict indoor PM2.5 in British Columbia, Canada. ACS EST Air. 2025;2:73–89.

[40] deSouza P, Kinney PL. On the distribution of low-cost PM 2.5 sensors in the US: demographic and air quality associations. J Expo Sci Environ Epidemiol. 2021. 31, 514–524. 10.1038/s41370-021-00328-2 33958706 10.1038/s41370-021-00328-2

[41] Mullen C, Flores A, Grineski S, Collins T. Exploring the distributional environmental justice implications of an air quality monitoring network in Los Angeles County. Environ Res. 2022;206:112612. 34953883 10.1016/j.envres.2021.112612

[42] Considine EM, Hao J, deSouza P, Braun D, Reid CE, Nethery RC. Evaluation of model-based PM2.5 estimates for exposure assessment during wildfire smoke episodes in the Western U.S. Environ Sci Technol. 2023. 10.1021/acs.est.2c06288. 36693177 10.1021/acs.est.2c06288 PMC10288567

[43] Orr A, Adam CE, Graham J, et al. A State of the Science Review of Wildfire-Specific Fine Particulate Matter Data Sources, Methods, and Models. Environ Health Perspect. 2025;133:066001. 40324008 10.1289/EHP15672 PMC12156208

[44] Qiu M, Kelp M, Heft-Neal S, Jin X, Gould CF, Tong DQ, et al. Evaluating chemical transport and machine learning models for wildfire smoke PM2.5: implications for assessment of health impacts. Environ Sci Technol. 2024. 10.1021/acs.est.4c05922. 39694472 10.1021/acs.est.4c05922

[45] Gan RW, Ford B, Lassman W, Pfister G, Vaidyanathan A, Fischer E, Volckens J, Pierce JR, Magzamen S. Comparison of wildfire smoke estimation methods and associations with cardiopulmonary-related hospital admissions. Geohealth. 2017;1:122–36. 28868515 10.1002/2017GH000073 PMC5580836

[46] Henderson SB, Brauer M, Macnab YC, Kennedy SM. Three measures of forest fire smoke exposure and their associations with respiratory and cardiovascular health outcomes in a population-based cohort. Environ Health Perspect. 2011;119:1266–71. 21659039 10.1289/ehp.1002288 PMC3230386

[47] Klepeis NE, Nelson WC, Ott WR, Robinson JP, Tsang AM, Switzer P, et al. The National Human Activity Pattern Survey (NHAPS): a resource for assessing exposure to environmental pollutants. J Expo Sci Environ Epidemiol. 2001;11:231–52. 10.1038/sj.jea.7500165 11477521

[48] Shrestha PM, Humphrey JL, Carlton EJ, Adgate JL, Barton KE, Root ED, et al. Impact of outdoor air pollution on indoor air quality in low-income homes during wildfire seasons. Int J Environ Res Public Health. 2019. 10.3390/ijerph16193535. 31546585 10.3390/ijerph16193535 PMC6801919

[49] Holder AL, Vreeland H, Brittingham H, Coefield S, Hassett-Sipple B, Deckmejian L, et al. Influence of building characteristics on wildfire smoke impacts on indoor air quality. ACS EST Air. 2025;2:1770–83.

[50] Sklar R, Picciotto S, Meltzer D, Goin DE, Huang S, Lurmann F, et al. Exploring relationships between smoke exposure, housing characteristics, and preterm birth in California. Environ Pollut. 2024. 10.1016/j.envpol.2024.125022. 39343350 10.1016/j.envpol.2024.125022 PMC13065353

[51] Ahn Y, Uejio CK, Wong S, Powell E, Holmes T. Spatial disparities in air conditioning ownership in Florida, United States. J Maps. 2023;19:2253262.

[52] Romitti Y, Sue Wing I, Spangler KR, Wellenius GA. Inequality in the availability of residential air conditioning across 115 US metropolitan areas. PNAS Nexus. 2022;1:pgac210. 36714868 10.1093/pnasnexus/pgac210 PMC9802221

[53] Krebs B, Neidell M. Wildfires exacerbate inequalities in indoor pollution exposure. Environ Res Lett. 2024;19:024043.

[54] Burke M, Heft-Neal S, Li J, et al. Exposures and behavioural responses to wildfire smoke. Nat Hum Behav. 2022;6:1351–61. 35798884 10.1038/s41562-022-01396-6

[55] Martenies SE, Wilson A, Hoskovec L, Bol KA, Burket TL, Podewils LJ, et al. The COVID-19-wildfire smoke paradox: reduced risk of all-cause mortality due to wildfire smoke in Colorado during the first year of the COVID-19 pandemic. Environ Res. 2023;225:115591. 36878268 10.1016/j.envres.2023.115591 PMC9985917

[56] Prichard SJ, O’Neill SM, Eagle P, Andreu AG, Drye B, Dubowy J, et al. Wildland fire emission factors in North America: synthesis of existing data, measurement needs and management applications. Int J Wildl Fire. 2020;29:132–47.

[57] Thurston GD, Ito K, Mar T, et al. Workgroup Report: Workshop on Source Apportionment of Particulate Matter Health Effects—Intercomparison of Results and Implications. Environ Health Perspect. 2005;113:1768–74. 16330361 10.1289/ehp.7989 PMC1314918

[58] (2019) Integrated Science Assessment (ISA) for Particulate Matter (Final US EPA NCFEA, Report. Dec 2019). In: US EPA. https://assessments.epa.gov/risk/document/&deid%3D347534. Accessed 30 July 2025.

[59] Aguilera R, Corringham T, Gershunov A, Benmarhnia T. Wildfire smoke impacts respiratory health more than fine particles from other sources: observational evidence from Southern California. Nat Commun. 2021;12:1493. 33674571 10.1038/s41467-021-21708-0 PMC7935892

[60] Li J, Cai YS, Kelly FJ, Wooster MJ, Han Y, Zheng Y, et al. Landscape fire smoke enhances the association between fine particulate matter exposure and acute respiratory infection among children under 5 years of age: findings of a case-crossover study for 48 low- and middle-income countries. Environ Int. 2023;171:107665. 36493611 10.1016/j.envint.2022.107665

[61] Zhang Y, Xu R, Huang W et al. Short-term exposure to wildfire-specific PM2.5 and hospitalization for diabetes morbidity: A study in multiple countries and territories. Diabetes Care. 2024; dc240703. 10.2337/dc24-0703 39012781

[62] Elser H, Frankland TB, Chen C, Tartof SY, Mayeda ER, Lee GS, Northrop AJ, Torres JM, Benmarhnia T, Casey JA. Wildfire smoke exposure and incident dementia. JAMA Neurol. 2024; e244058. 10.1001/jamaneurol.2024.4058 PMC11589856 39585704

[63] Yu W, Lall R, Thurston G. Comparing the cardiopulmonary health implications of wildfire vs. non-wildfire PM2.5 particles in New York City. Am J Respir Crit Care Med. 2025;211:A5406–A5406.

[64] Briggs NL, Jaffe DA, Gao H, Hee JR, Baylon PM, Zhang Q, et al. Particulate matter, ozone, and nitrogen species in aged wildfire plumes observed at the Mount Bachelor Observatory. Aerosol Air Qual Res. 2016;16:3075–87.

[65] O’Dell K, Hornbrook RS, Permar W, et al. Hazardous air pollutants in fresh and aged Western US wildfire smoke and implications for long-term exposure. Environ Sci Technol. 2020. 10.1021/acs.est.0c04497. 32857515 10.1021/acs.est.0c04497

[66] Yu Y, Zou W, Jerrett M, Meng Y-Y. Acute health impact of wildfire-related and conventional PM2.5 in the United States: a narrative review. Environ Adv. 2023;12:100179.

[67] Riss CS, Faulstich SD, Reuther PS, Metcalf WJ, Darrow LA, Holmes HA, Strickland MJ. Influence of fire characteristics on the associations between smoke PM2.5 exposure and acute cardiorespiratory health events. Environ Int. 2025;201:109577. 40480103 10.1016/j.envint.2025.109577 PMC12208789

[68] Holder AL, Ahmed A, Vukovich JM, Rao V. Hazardous air pollutant emissions estimates from wildfires in the wildland urban interface. PNAS Nexus. 2023;2:pgad186. 37346272 10.1093/pnasnexus/pgad186 PMC10281377

[69] National Academies of Sciences, Engineering, and Medicine, Division on Earth and Life Studies, Board on Chemical Sciences and Technology, Committee on the Chemistry of Urban Wildfires. The Chemistry of Fires at the Wildland-Urban Interface. Washington (DC): National Academies Press (US); 2022. 36657007

[70] Higuera PE, Cook MC, Balch JK, Stavros EN, Mahood AL. St. Denis LA.Shifting social-ecological fire regimes explain increasing structure loss from Western wildfires. PNAS Nexus. 2023; pgad005. 10.1093/pnasnexus/pgad005 PMC10019760 36938500

[71] Sekimoto K, Coggon MM, Gkatzelis GI, Stockwell CE, Peischl J, Soja AJ, et al. Fuel-type independent parameterization of volatile organic compound emissions from Western US wildfires. Environ Sci Technol. 2023. 10.1021/acs.est.3c00537. 37611137 10.1021/acs.est.3c00537 PMC10483695

[72] Xu R, Ye T, Yue X, et al. Global population exposure to landscape fire air pollution from 2000 to 2019. Nature. 2023;621:521–9. 37730866 10.1038/s41586-023-06398-6 PMC10511322

[73] US EPA O. Integrated Science Assessment (ISA) for ozone and related photochemical oxidants. 2015;. https://www.epa.gov/isa/integrated-science-assessment-isa-ozone-and-related-photochemical-oxidants. Accessed 23 Aug 2025.

[74] Chen G, Guo Y, Yue X, et al. All-cause, cardiovascular, and respiratory mortality and wildfire-related ozone: a multicountry two-stage time series analysis. Lancet Planet Health. 2024;8:e452–62. 38969473 10.1016/S2542-5196(24)00117-7

[75] Reid CE, Considine EM, Watson GL, Telesca D, Pfister GG, Jerrett M. Associations between respiratory health and ozone and fine particulate matter during a wildfire event. Environ Int. 2019;129:291–8. 31146163 10.1016/j.envint.2019.04.033

[76] CDC. Safety Guidelines: Wildfires and Wildfire Smoke. In: Wildfires 2025;. https://www.cdc.gov/wildfires/safety/how-to-safely-stay-safe-during-a-wildfire.html. Accessed 4 Oct 2025.

[77] Nethery RC, Mealli F, Sacks JD, Dominici F. Evaluation of the health impacts of the 1990 Clean Air Act Amendments using causal inference and machine learning. J Am Stat Assoc. 2021;116:1128–39. 10.1080/01621459.2020.1803883 PMC7788006 33424062

